# Application of direct stochastic optical reconstruction microscopy (dSTORM) to the histological analysis of human glomerular disease

**DOI:** 10.1002/cjp2.217

**Published:** 2021-05-21

**Authors:** Edwin Garcia, Jonathan Lightley, Sunil Kumar, Ranjan Kalita, Frederik Gőrlitz, Yuriy Alexandrov, Terry Cook, Christopher Dunsby, Mark AA Neil, Candice A Roufosse, Paul MW French

**Affiliations:** ^1^ Photonics Group, Physics Department Imperial College London London UK; ^2^ Imperial College London Photonics Satellite Laboratory Francis Crick Institute London UK; ^3^ Department of Inflammation and Immunology Imperial College London London UK

**Keywords:** histopathology, super‐resolved microscopy, dSTORM, immunofluorescence, kidney, glomerulus

## Abstract

Electron microscopy (EM) following immunofluorescence (IF) imaging is a vital tool for the diagnosis of human glomerular diseases, but the implementation of EM is limited to specialised institutions and it is not available in many countries. Recent progress in fluorescence microscopy now enables conventional widefield fluorescence microscopes to be adapted at modest cost to provide resolution below 50 nm in biological specimens. We show that stochastically switched single‐molecule localisation microscopy can be applied to clinical histological sections stained with standard IF techniques and that such super‐resolved IF may provide an alternative means to resolve ultrastructure to aid the diagnosis of kidney disease where EM is not available. We have implemented the direct stochastic optical reconstruction microscopy technique with human kidney biopsy frozen sections stained with clinically approved immunofluorescent probes for the basal laminae and immunoglobulin G deposits. Using cases of membranous glomerulonephritis, thin basement membrane lesion, and lupus nephritis, we compare this approach to clinical EM images and demonstrate enhanced imaging compared to conventional IF microscopy. With minor modifications in established IF protocols of clinical frozen renal biopsies, we believe the cost‐effective adaptation of conventional widefield microscopes can be widely implemented to provide super‐resolved image information to aid diagnosis of human glomerular disease.

## Introduction

Kidney disease is highly complex and challenging to diagnose, typically requiring light microscopy (LM), immunohistology, and electron microscopy (EM). EM is useful in the histopathology of ~50% of native kidney biopsies and essential for the diagnosis of ~20% [1, 2, 3], making the use of EM a standard technique in many countries for native kidney biopsy examination following LM and immunofluorescence (IF) imaging. EM is required for the diagnosis of kidney diseases associated with structural abnormalities of the basement membrane (e.g. inherited abnormalities of collagen type IV alpha chains), diseases with fibrils (e.g. fibrillary and immunotactoid glomerulonephritides), and rare genetic diseases such as Fabry's disease or lecithin cholesterol acyl transferase deficiency. It is also routinely used to document morphological changes in podocytes, and to document shape, substructure, and position relative to the glomerular basement membrane (GBM) of immune complexes and/or complement fragment deposits.

However, EM instrumentation is not available to much of the world's population and the number of expert clinical EM staff and facilities is decreasing where it is available. For both developing and developed countries, it would be useful to find a cheaper alternative to EM to enhance diagnosis beyond what is possible with LM and IF, and it would be useful to simplify and accelerate the diagnostic workflow by reducing the number of instruments required. Recently, optical microscopy has been extended below the diffraction limit with super‐resolved microscopy (SRM) techniques such as structured illumination microscopy (SIM) approaches [4, 5], stochastically switched single‐molecule localisation microscopy (SMLM) techniques such as photo‐activated localisation microscopy [6, 7] and stochastic optical reconstruction microscopy (STORM) [8], and RESOLFT [9] techniques such as stimulated emission depletion microscopy [10, 11]. Of these SRM techniques, SMLM approaches have the simplest requirements for instrumentation – making them cost‐effective and potentially accessible to a broad spectrum of laboratories – and can provide spatial resolution below 50 nm, which approaches that of EM. SMLM utilises sequential emission and localisation of stochastically ‘blinking’ fluorophores that are sufficiently sparse at any given time to permit the position of each emitter to be determined with high precision by determining the centre of the recorded intensity distribution. This emitter blinking can be realised in many ways, e.g. by photoswitching fluorophores to emit in the detection band or otherwise, by photobleaching to terminate emission or by utilising appropriate chemical buffers to facilitate reversible photoswitching of fluorophores in and out of dark states, as demonstrated in the technique described as direct STORM (dSTORM) [12]. In previous work developing an approach we described as ‘easySTORM’, we have shown that dSTORM can be robustly implemented at a relatively low cost (<£20,000) using multimode diode lasers and multimode optical fibres to provide super‐resolved images over large (>120 × 120 μm) fields of view [13]. We here show that this approach can be applied to clinical histological sections to provide super‐resolved IF imaging using clinically approved antibodies – an approach we describe as ‘histo*STORM*’.

We specifically explore the potential to replace EM with histo*STORM* in the diagnosis of kidney disease and to potentially provide a widely accessible clinical tool based on much lower cost instrumentation. This follows earlier work using SIM [14] and STORM [15] to study renal podocyte substructure and protein organisation in the GBM. Although this prior work demonstrated the potential of super‐resolved IF, it was realised with expensive commercial SRM instrumentation and the study utilising STORM was undertaken with mouse tissue and non‐clinically approved antibodies. We aim to develop a low‐cost approach that could be accessible by clinicians in low‐ and middle‐income countries by utilising ‘easySTORM’ to image clinically relevant proteins, such as immunoglobulin G (IgG) in GBM, with existing clinically validated antibodies and to develop practical protocols to work with existing biopsy samples such as frozen sections or formalin‐fixed paraffin‐embedded (FFPE) sections. We note that STORM has previously been applied to research pathology, e.g. to study epigenetic modulation [16] and the progression of cancer [17], but not to clinical histological sections using clinically approved antibodies.

## Materials and methods

### Sample preparation

The protocol to prepare kidney biopsies for histoSTORM is provided in Table 1. Frozen kidney biopsy sections of 3 μm thickness on slides were circumscribed with the addition of silicon (Polycraft ZA22 Mould RTV Addition Cure Mould Making Silicone Rubber; MB Fibreglass, Newtownabbey, Northern Ireland) and polymerised at room temperature to achieve rubber consistency and form a well of 0.1 ml volume. For FFPE sections, paraffin was removed with xylene, using two treatments for 5 min and then ethanol washes of 2 min with decreasing ethanol dilutions in water at 100, 75, 50, 25, and 0%. Samples were allowed to dry and additional silicon was applied to reinforce the sample well. Acetone fixation for 10 min followed by three quick washes with phosphate‐buffered saline (PBS) was followed by antigen retrieval at 37 °C for 27 min with 4 ml of Protease Type 24 (P8038; Sigma‐Aldrich, Dorset, UK) at 0.125 mg/ml in prewarmed PBS covering the whole slide. Slides were then washed three times for 5 min at room temperature in a Coplin jar with 50 ml of diluted PBS (pH 7.4) in water at a ratio of 1:10. Samples were then incubated for 10 min in 0.1 ml of PBS with 1 mg/ml sodium borohydride to reduce tissue autofluorescence, followed by three further washes as previously described. Unspecific antibody‐binding sites were reduced on samples by blocking with 0.1 ml of 3% (w/v) bovine serum albumin (BSA) in PBS at room temperature for 10 min.

**Table 1:** Sample preparation for dSTORM of fixed kidney histological sections.

**Antigen retrieval**	
Protease Type 24 (P8038 – Sigma‐Aldrich) (0.125 mg/ml) in PBS	37 °C for 27 min
Wash in 50 ml PBS 3 times in a Coplin jar	5 min each
**Autofluorescence quenching**	
0.1 ml sodium borohydride (1 mg/ml in PBS)	10 min
**Blocking**	
0.1 ml 3% BSA in PBS	10 min
**Primary probes**	
Primary antibody cocktail 0.1 ml (3% BSA in PBS) Laminin (MAB1920 – Millipore) and IgG (A0423 – Dako) or rabbit isotype (X0936 – Dako). Diluted 1:10,000 in 3% BSA	20 min
Wash in 50 ml PBS 3 times in a Coplin jar	5 min each
**Secondary probes**	
Secondary antibody cocktail 0.1 ml (3% BSA in PBS) (goat anti‐rabbit IgG H+L) 0.25 mg/ml (16837 – AAT Bioquest) (goat anti‐mouse IgG H+L) 1:2,000 (A32727 – Invitrogen)	20 min
Wash in 50 ml PBS 3 times in a Coplin jar	5 min each
**Post fixation**	
0.1 ml acetone	5 min
Wash quickly in PBS diluted 1:10 in water	3x
**Tissue clearing**	
0.1 ml of 70% TDE in PBS	10 min
**Sample preparation in STORM buffer**	
0.1 ml of STORM buffer with 60% TDE (mercaptoethylamine 50 mm, d‐lactate 10 mm, and 60% TDE in PBS and 0.75 U/ml of Oxyrase‐EC [SAE0010 – Sigma‐Aldrich])	30 min
Mount slide in fresh STORM buffer with 60% TDE	

The tissue samples were then labelled using 0.1 ml of a cocktail of primary antibodies – Laminin MAB1920 (Millipore, Watford, Hertfordshire, UK) and IgG (A0423; Agilent Dako, Stockport, Cheshire, UK) or Rabbit Isotype X0936 (Agilent Dako) – diluted at 1:10,000 ratio in 3% (w/v) BSA at room temperature for 20 min. The samples were then washed three times as previously described and treated with 0.1 ml of a cocktail of secondary antibodies – goat anti‐mouse IgG H+L diluted at 1:2,000 ratio (A32727; Invitrogen, ThermoFisher Scientific, Loughborough, Leicestershire, UK) and 0.25 mg/ml goat anti‐rabbit IgG H+L (16837; AAT Bioquest, Stratech, Ely, Cambridgeshire, UK) diluted at 1:2,000 ratio in 3% BSA at room temperature for 20 min. The secondary antibodies were conjugated to either Alexa Fluor 555 (ThermoFisher Scientific) or iFluor 647 (AAT Bioquest). The samples were then washed a further three times and then fixed with acetone for 5 min.

To improve image quality, the tissues were then chemically cleared by immersing in 0.1 ml of 70% 2′2 thiodiethanol (TDE) (166782; Sigma‐Aldrich) in PBS for 10 min at room temperature [16]. Samples were then treated with 0.1 ml of STORM buffer (mercaptoethylamine 50 mm, d‐lactate 10 mm, and 60% TDE in PBS and 0.75 U/ml of Oxyrase‐EC [SAE0010; Sigma‐Aldrich]) at room temperature for 30 min. Immediately prior to imaging, the sample was mounted in a fresh preparation of STORM buffer.

### Imaging

We employed our easySTORM [13] implementation of dSTORM that utilises a set of fibre‐coupled multimode laser diode sources (Laserbank; Cairn Research Ltd, Faversham, Kent, UK) with a standard inverted microscope frame (Axiovert 200; Carl Zeiss GmbH, Jena, Germany) and an excitation beam coupling unit (OptoTIRF; Cairn Research Ltd) that can be configured for epifluorescence or total internal reflection. We note that these commercial components can be replaced with much lower cost home‐built equivalents [18] and a complete super‐resolved IF microscope can be assembled for a component cost less than £20,000 [18]. For the results presented here, dSTORM of the histological sections was undertaken using a ×100, 1.46 numerical aperture oil lens in the epifluorescence microscope with an sCMOS camera (Photometrics Prime 95B, Tucson, AZ, USA).

To image structures labelled with Alexa Fluor 555, excitation at 520 nm was initially set to 1,045 μW/cm^2^ for 5–10 s to activate fluorophore blinking and then the power was decreased by ~50% during dSTORM image acquisition. To image structures labelled with iFluor 647, excitation at 635 nm was initially set to 2,500 μW/cm^2^ at the sample plane for 5–10 s and then reduced by ~50% during dSTORM image acquisition The camera integration time was set to 30 ms, with images acquired at a frame rate of 33 Hz.

Super‐resolved images were reconstructed by ThunderSTORM with drift correction enabled, as reported previously [13]. Two‐channel images were generated with a cross‐correlation function that can be found at https://github.com/yalexand/Imperial-ClusDoC.git on the Alexa Fluor 555 and iFluor 647 channels.

## Results and discussion

In this initial small study, we applied histoSTORM with standard clinically approved antibodies to both FFPE and frozen histological sections and, while we expect the sample processing protocols can be further optimised, we believe that the exemplar results presented below show ultrastructure of clinical interest that could potentially aid diagnosis.

**Figure 1:**
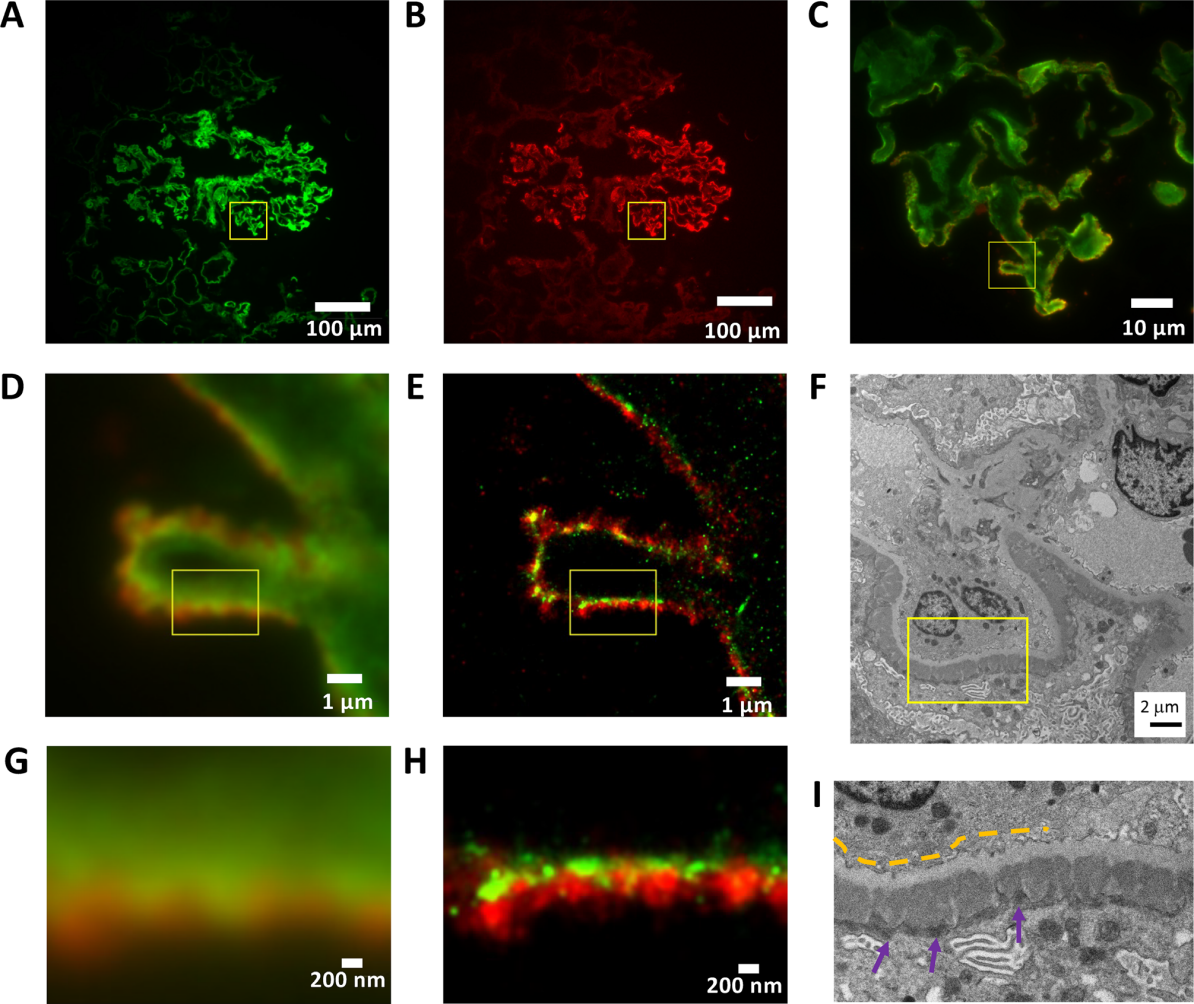
Membranous glomerulonephritis. Basement membrane (laminin, green – Alexa Fluor 555) and IgG deposits (red – iFluor 647). (A–C) Widefield IF images at ×100 magnification of frozen section of membranous glomerulonephritis showing (A) laminin channel, (B) IgG channel, and (C) expanded two‐channel image of region indicated by yellow square in (A) and (B). (D) Widefield IF of region indicated by yellow square in (C), and (E) corresponding STORM image with pixel size rendered at 25 nm. (F) Electron micrograph of similar structure from same biopsy at ×5,500 magnification. (G) Widefield IF image of 3.2 × 2.4 μm^2^ region indicated in (D) and (E), with (H) corresponding STORM image. (I) Expanded electron micrograph image of region indicated in (F). Yellow dashed lines indicate the light grey GBM. Dark grey electron‐dense deposits on the subepithelial side (purple arrows) represent immune complexes containing IgG.

Membranous glomerulonephritis (Figure 1) is characterised by subepithelial immune complex deposits containing IgG in the GBM with thickening of this structure [19]. The two‐colour widefield IF image in Figure 1D shows a capillary loop with the GBM in green stained with an anti‐human laminin probe (green, Alexa Fluor 555) and IgG deposits on the epithelial side of the filtration barrier in red (IgG, iFluor 647). dSTORM images rendered at 25 nm per pixel reveal well‐defined subepithelial deposits that are consistent with those observed by EM (Figure 1F,I). Whilst there is no clear definition of structures in the widefield IF images (Figure 1D,G), details of immune deposits at subepithelial regions and a gradient of content of immune deposits are readily observed in dSTORM (Figure 1E,H). Green regions of the dSTORM images indicate areas of GBM free of immune deposits, yellow areas indicate overlap of laminin and immune deposits, and red areas indicate clusters of immune deposits.

**Figure 2:**
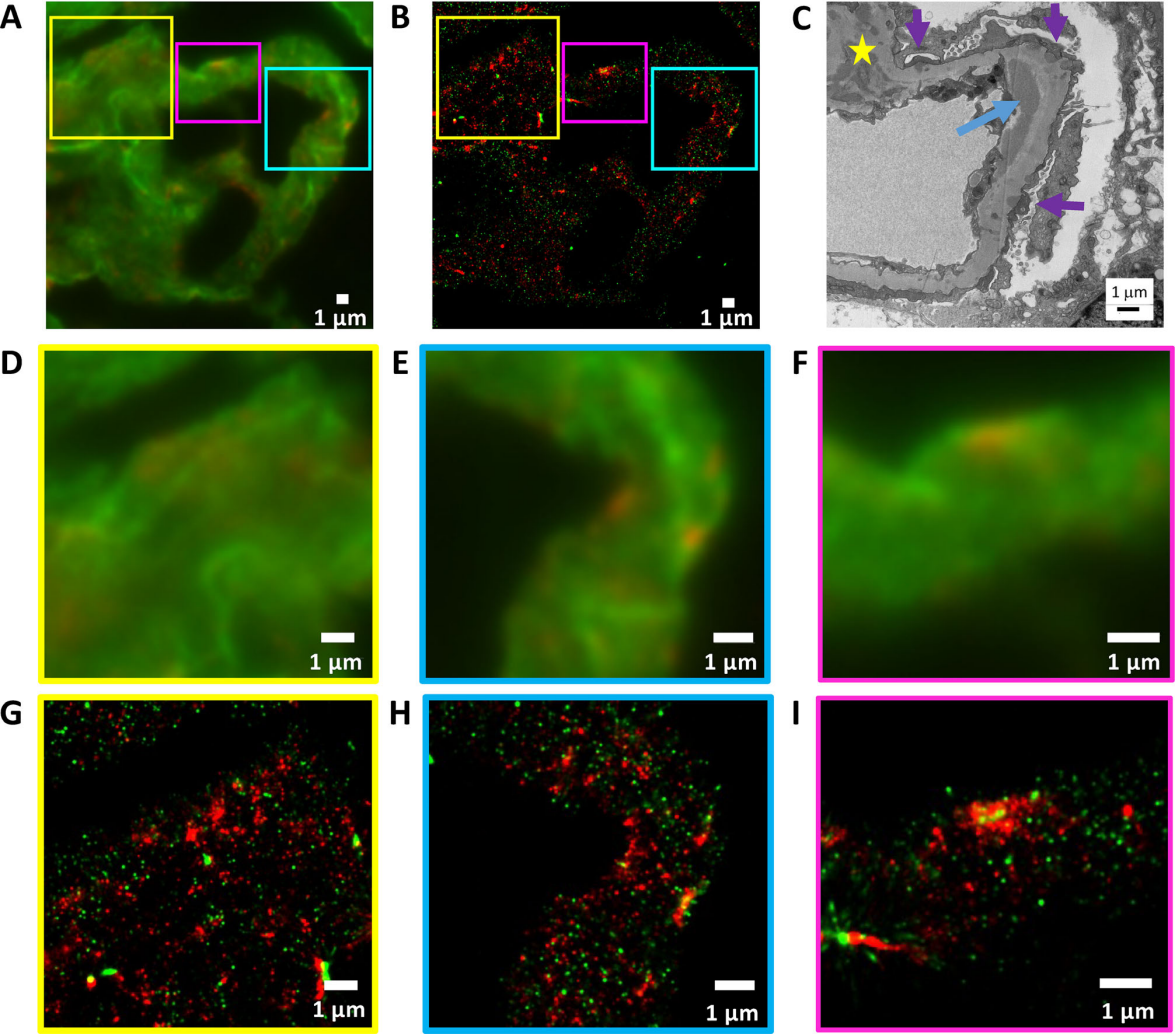
Lupus nephritis type IV. Basement membrane (laminin, green – Alexa Fluor 555) and IgG deposits (red –iFluor 647). (A) Widefield IF image at ×100 magnification of frozen section showing lupus nephritis type IV with selected regions presenting (D,G) mesangial deposits, (E,H) subendothelial deposits, and (F,I) subepithelial deposits. (B) STORM image rendered with pixel size of 25 nm corresponding to (A). (C) Electron micrograph of a similar structure from the same sample at ×8,000 magnification, presenting occasional electron‐dense deposits containing IgG on the subepithelial side of the GBM (purple arrows), on the subendothelial side of the GBM (blue arrow), and in the mesangium (yellow star). (G–I) STORM images corresponding to widefield IF images (D–F). (D) and (G) show the region indicated by the yellow square in (A) and (B). (E) and (H) show the region indicated by the cyan square in (A) and (B). (F) and (I) show the region indicated by the purple square in (A) and (B).

Lupus nephritis is characterised by glomerular deposition of polyclonal IgG in various areas of the glomerulus [20, 21]. Figure 2 shows deposits of IgG (red, iFluor 647) and basement membrane staining (laminin, green, Alexa Fluor 555) in a glomerular capillary of stage IV lupus nephritis. In this stage, mesangial (Figure 2G), subendothelial (Figure 2H), and subepithelial (Figure 2I) IgG deposits are readily observed with dSTORM and recapitulate the distribution of high electron density IgG deposits documented with EM (Figure 2C).

**Figure 3:**
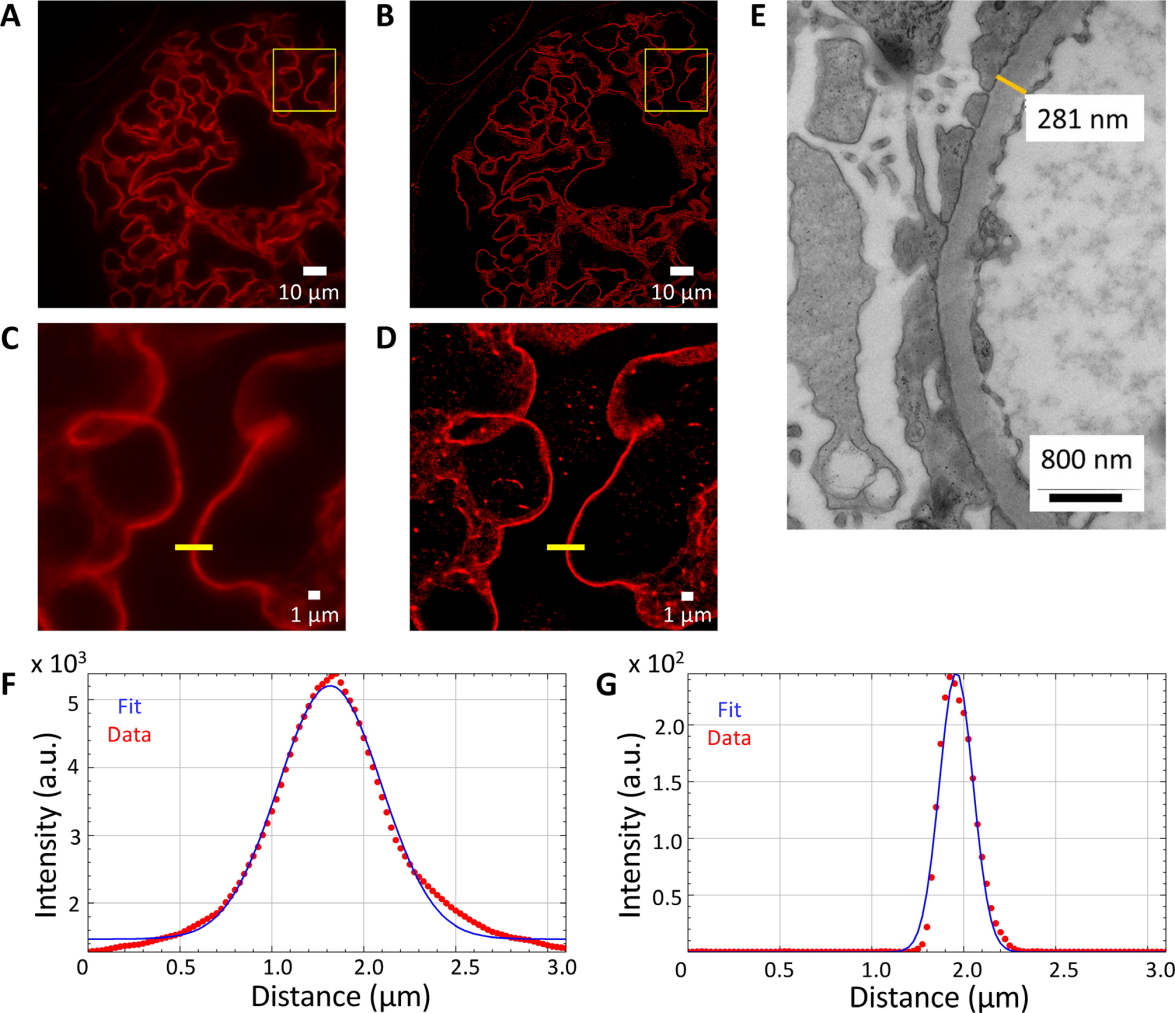
Minimal change disease: GBM thickness measurements (Laminin‐iFluor 647). (A) Widefield IF image at ×100 magnification of FFPE section. (B) Rendered STORM image of region shown in (A). (C) Widefield inset of the region shown in the yellow box in (A). (D) STORM inset of the region shown in the yellow box in (B) rendered with a pixel size of 25 nm. (E) Electron micrograph of a GBM from a different section of the same biopsy at ×15,500 magnification, for which the GBM thickness at the indicated position is 281 nm. (F) Measured thickness (full width at half maximum) of GBM from widefield IF image (C) at the position of the yellow line (657 nm). (G) Measured thickness (full width at half maximum) of STORM image (D) at the position of the yellow line (212 nm).

EM is also routinely used to measure the thickness of the GBM [22]. The large field of view (of the order of 120 μm × 120 μm) with resolution below diffraction limit provided by the easySTORM platform allows evaluation of GBM thickness in glomerular capillaries and documentation of other aspects of the glomerulus. Figure 3A,C shows widefield epifluorescence images of an FFPE section from a biopsy of minimal change disease, where the laminin in the GBM is labelled with iFluor 647. Figure 3B,D shows the corresponding STORM images and the ability of the STORM images to enable GBM thickness measurements below the diffraction limit is confirmed. Figure 3F,G shows line sections through the widefield and STORM images of the GBM. Figure 3E shows an electron micrograph with GBM width of 281 nm.

In conclusion, we have demonstrated that histoSTORM of frozen or FFPE kidney biopsy sections can provide additional information compared to conventional widefield IF. histoSTORM can precisely locate subepithelial, subendothelial, and mesangial immune complex deposits, which can aid the diagnosis of glomerulonephritis, and enables the thickness of the GBM to be measured with sufficient resolution to aid diagnostic assessments where EM is not available. We note that recalibration of the thickness of the GBM measured using histoSTORM relative to that using EM may be required to establish diagnostic criteria.

As well as being much cheaper to implement and sustain, compared to EM, the sample preparation for histoSTORM is similar to IF and the larger fields of view enable much faster imaging than EM. This could allow, for example, multiple capillaries to be routinely documented within a glomerulus, including at multiple planes along specimen depth. We note that emerging image processing tools, including those based on machine learning, could further enhance the ability of histoSTORM to probe ultrastructure and to diagnose disease.

While this initial study does not establish that histoSTORM can fully replace EM in renal diagnosis, it does provide evidence of added value relative to LM and IF. However, further prospective studies of large case series would be required to establish its clinical utility. histoSTORM could be useful as an auxiliary technique – as could other advanced optical microscopy techniques such as SIM [14] – and could refine current classification stages of glomerular lesions and other renal pathologies, noting that these are periodically revisited and modified based on new findings and progress in understanding of mechanisms of disease and tissue injury [20, 21]. There may also be a clinical role for correlative STORM/EM, as previously presented in mouse kidney frozen sections [15].

Ultimately, histoSTORM may not be able to replace EM for all renal diagnoses but we believe that it has the potential for wide clinical impact, especially in less well‐resourced settings where EM is not available.

## Author contributions statement

CAR, TC, CD and PMWF conceived the idea and application. EG and CAR prepared samples. EG, JL, RK and SK acquired images. SK, FG, JL, MAAN, CD and PMWF developed the instrument. EG, YA and MAAN analysed the data. CAR interpreted images.

## References

[1] Haas M. A reevaluation of routine electron microscopy in the examination of native renal biopsies. J Am Soc Nephrol 1997; 8: 70–76. 9013450 10.1681/ASN.V8170

[2] Shore I, Moss J. Electron microscopy in diagnostic renal pathology. Curr Diagn Pathol 2002; 8: 207–215.

[3] Kurien AA, Larsen C, Rajapurkar M, *et al*. Lack of electron microscopy hinders correct renal biopsy diagnosis: a study from India. Ultrastruct Pathol 2016; 40: 14–17. 26771449 10.3109/01913123.2015.1120837

[4] Gustafsson MGL. Surpassing the lateral resolution limit by a factor of two using structured illumination microscopy. J Microsc 2000; 198: 82–87. 10810003 10.1046/j.1365-2818.2000.00710.x

[5] Gustafsson MGL. Nonlinear structured‐illumination microscopy: wide‐field fluorescence imaging with theoretically unlimited resolution. Proc Natl Acad Sci U S A 2005; 102: 13081–13086. 16141335 10.1073/pnas.0406877102 PMC1201569

[6] Betzig E, Patterson GH, Sougrat R, *et al*. Imaging intracellular fluorescent proteins at nanometer resolution. Science 2006; 313: 1642–1645. 16902090 10.1126/science.1127344

[7] Hess ST, Girirajan TPK, Mason MD. Ultra‐high resolution imaging by fluorescence photoactivation localization microscopy. Biophys J 2006; 91: 4258–4272. 16980368 10.1529/biophysj.106.091116 PMC1635685

[8] Rust MJ, Bates M, Zhuang X. Sub‐diffraction‐limit imaging by stochastic optical reconstruction microscopy (STORM). Nat Methods 2006; 3: 793–796. 16896339 10.1038/nmeth929 PMC2700296

[9] Hofmann M, Eggeling C, Jakobs S, *et al*. Breaking the diffraction barrier in fluorescence microscopy at low light intensities by using reversibly photoswitchable proteins. Proc Natl Acad Sci U S A 2005; 102: 17565–17569. 16314572 10.1073/pnas.0506010102 PMC1308899

[10] Hell SW, Wichmann J. Breaking the diffraction resolution limit by stimulated emission: stimulated‐emission‐depletion fluorescence microscopy. Opt Lett 1994; 19: 780–782. 19844443 10.1364/ol.19.000780

[11] Klar TA, Jakobs S, Dyba M, *et al*. Fluorescence microscopy with diffraction resolution barrier broken by stimulated emission. Proc Natl Acad Sci U S A 2000; 97: 8206–8210. 10899992 10.1073/pnas.97.15.8206 PMC26924

[12] Heilemann M, van de Linde S, Schüttpelz M, *et al*. Subdiffraction‐resolution fluorescence imaging with conventional fluorescent probes. Angew Chem Int Ed 2008; 47: 6172–6176. 10.1002/anie.200802376 18646237

[13] Kwakwa K, Savell A, Davies T, *et al*. easySTORM: a robust, lower‐cost approach to localisation and TIRF microscopy. J Biophotonics 2016; 9: 948–957. 27592533 10.1002/jbio.201500324

[14] Pullman JM, Nylk J, Campbell EC, *et al*. Visualization of podocyte substructure with structured illumination microscopy (SIM): a new approach to nephrotic disease. Biomed Opt Express 2016; 7: 302–311. 26977341 10.1364/BOE.7.000302 PMC4771450

[15] Suleiman H, Zhang L, Roth R, *et al*. Nanoscale protein architecture of the kidney glomerular basement membrane. Elife 2013; 2: e01149. 24137544 10.7554/eLife.01149 PMC3790497

[16] Xu J, Ma H, Jin J, *et al*. Super‐resolution imaging of higher‐order chromatin structures at different epigenomic states in single mammalian cells. Cell Rep 2018; 24: 873–882. 30044984 10.1016/j.celrep.2018.06.085 PMC6154382

[17] Xu J, Ma H, Ma H, *et al*. Super‐resolution imaging reveals the evolution of higher‐order chromatin folding in early carcinogenesis. Nat Commun 2020; 11: 1899. 32313005 10.1038/s41467-020-15718-7 PMC7171144

[18] Biophotonics Research Group, Imperial College London . [Accessed 26 March 2021]. Available from: https://www.imperial.ac.uk/photonics/research/biophotonics/instruments–software/super-resolved-microscopy/easystorm/

[19] Lai WL, Yeh TH, Chen PM, *et al*. Membranous nephropathy: a review on the pathogenesis, diagnosis, and treatment. J Formos Med Assoc 2015; 114: 102–111. 25558821 10.1016/j.jfma.2014.11.002

[20] Weening JJ, D'Agati VD, Schwartz MM, *et al*. The classification of glomerulonephritis in systemic lupus erythematosus revisited. J Am Soc Nephrol 2004; 15: 241–250. 14747370 10.1097/01.asn.0000108969.21691.5d

[21] Haas M, Seshan SV, Barisoni L, *et al*. Consensus definitions for glomerular lesions by light and electron microscopy: recommendations from a working group of the Renal Pathology Society. Kidney Int 2020; 98: 1120–1134. 32866505 10.1016/j.kint.2020.08.006

[22] Tryggvason K, Patrakka J. Thin basement membrane nephropathy. J Am Soc Nephrol 2006; 17: 813–822. 16467446 10.1681/ASN.2005070737

